# Effect of vitamin D3 supplementation on thyroid function of clinically healthy dogs

**DOI:** 10.3389/fvets.2025.1559608

**Published:** 2025-10-10

**Authors:** S. Hashemi, T. Shomali, N. Derakhshandeh, S. Nazifi

**Affiliations:** ^1^Division of Pharmacology and Toxicology, Department of Basic Sciences, School of Veterinary Medicine, Shiraz University, Shiraz, Iran; ^2^Department of Clinical Sciences, School of Veterinary Medicine, Shiraz University, Shiraz, Iran

**Keywords:** vitamin D, thyroid hormones, thyrotropin, dog, FT3/FT4 ratio

## Abstract

**Introduction:**

Vitamin D has diverse effects on different organ functions. This study evaluated the effect of vitamin D3 supplementation on thyroid function of healthy dogs by repeated assays of thyroid hormones (including total and free T4 and T3) as well as TSH levels during a 6 week-period.

**Methods:**

Eight healthy adult male dogs received vitamin D3 supplements at the dose of 50 IU/kg BW per day. Venous blood samples were collected on days 0, 14, 28 and 42 of the experiment.

**Results:**

Six-week vitamin D3 supplementation statistically increased serum T4 levels [*F* (1.89, 13.2) =8.39 and *p* = 0.004]. Serum T4 levels on days 28 and 42 were significantly higher than the baseline (day 0) (*p* < 0.05). There was also a statistically significant effect of duration of supplementation on serum fT4 levels, [*F* (1.63, 11.4) = 12.53, *p* = 0.014], although the difference was only significant between days 0 and 42. Changes in serum levels of T3 and fT3 were non-significant. TSH levels showed a significant decrease during the whole time of the study [*F* (1.17, 7.02) = 26.4 and *p* = 0.001]. On days 14, 28 and 42 this parameter was statistically lower than day 0. Changes in serum T3/T4 during time were not statistically significant. However; the fT3/fT4 ratio showed a downward change during study [*F* (1.77, 8.87) = 5.18 and *p* = 0.035]. The fT3/fT4 ratio on day 42 of the experiment was significantly lower than day 0.

**Discussion:**

Vitamin D3 supplementation to healthy dogs is associated with a time-dependent change in thyroid hormone profile (increased serum T4 and fT4) which are probably mediated at the thyroid gland level as shown by the negative feedback on serum TSH concentrations. These findings pave the road for future studies on the plausible effects of this vitamin on thyroid function of hypothyroid dogs.

## Introduction

Thyroid gland is a major endocrine organ which secrets thyroid hormones, most importantly thyroxine (T4) and triiodothyronine (T3), that play a critical role in regulation of basal metabolic rate. Only less than 1% of the thyroid hormones is unbound to plasma proteins and therefore is active. Thyroid hormone affinity for plasma proteins in dogs is lower than humans. As a result, dogs have lower serum total thyroid hormone concentration and higher free hormone concentrations compared to humans (1). The function of the thyroid gland is regulated by the hypothalamic–pituitary-thyroid axis where free thyroid hormones have a negative feedback effect on the thyrotropin (TSH) secretion (2).

Hypothyroidism is an important common endocrinopathy of dogs. In about 95% of cases, there is a primary hypothyroidism with lymphocytic thyroiditis and idiopathic follicular atrophy as the two most important pathological features (3).

Vitamin D is well-known for its important roles in the homeostasis of calcium and phosphorus and maintenance of bone health (4). However, relatively recent evidence demonstrates that this vitamin has diverse effects on different body systems including thyroid gland (5). Vitamin D deficiency is a risk factor for many thyroid disorders, including autoimmune thyroid diseases and thyroid cancer in humans (6). Studies on humans that have addressed the effects of vitamin D supplementation on total and free thyroid hormones and TSH levels in different conditions have shown contradictory results (7). A recent systematic review examining the impact of vitamin D supplementation on thyroid function in individuals with autoimmune thyroid disease found that short-term studies (lasting three months or less) did not show notable differences in average levels of TSH, T3, or T4 when compared to the control group. In contrast, all long-term studies (longer than three months) demonstrated significant enhancements in mean TSH, T3, and T4 levels compared to the control group. Furthermore, when baseline measurements were utilized for comparison, every long-term study indicated significant changes in TSH, T3, and T4 by the end of the trials (8).

Dogs are not able to efficiently synthesize vitamin D when exposed to sunlight, therefore; acquiring sufficient amount of this vitamin from diet is very important in this species (9). Like humans, different disease states have been associated with low serum vitamin D levels in dogs (10) and vitamin D deficiency also occurs in dogs due to consumption of low quality diets (11). As a fat-soluble vitamin, vitamin D has a long whole-body half-life of about 2 months. Half-lives for 25-hydroxy vitamin D and 1,25-dihydroxy vitamin D are about 2 to 3 weeks and 4 to 6 h, respectively (12).

In a previous study, we reported that supplementation of healthy dogs with 50 IU/kg BW of vitamin D3 daily can increase serum levels of 25-hydroxy vitamin D without adverse effects on liver and kidney parameters (13). Regarding the fact that knowledge on the effect of vitamin D supplementation on thyroid function of dogs is very scarce, in this study we evaluated the effect of vitamin D3 supplementation on serum thyroid hormone levels (including total and free T4 and T3) as well as TSH levels in healthy dogs.

## Materials and methods

### Study design

A total of eight intact male adult dogs, aged 2 to 3 years and of mixed breeds were included in the study. Dogs were owned by our school. The average body weight of the dogs was 20 kg, ranging from 19 to 22 kg, and their body condition scores ranged from 3 to 4 on a 9-point scale. The dogs were vaccinated against canine distemper virus (CDV), canine adenovirus (CAV; type 2) and canine parvovirus type 2 (CPV-2) and its variants as well as rabies virus, canine parainfluenza virus and *Leptospira* spp. (with serogroups icterohaemorrhagiae, grippotyphosa and sejroe) based on 2016 WSAVA guidelines for the vaccination of dogs and cats (14). The whole study was performed during winter (January and February). The dogs were in good health and underwent a 2-week acclimation period prior to the commencement of the experiment.

Dogs were healthy in clinical examination and also based on the results of blood work (complete blood count (CBC) with differential as well as routine liver and kidney parameters).

Dogs were housed in individual pens and provided with 300 g of dry dog food per 20 kg of body weight once daily, as recommended by the manufacturer (Adult Nutripet dry dog food for dogs with moderate physical activity, Behintash Co., Tehran, Iran), along with tap water available constantly. The food contained 2,900 IU/kg dry matter of vitamin D3 which was compatible with the National Research Council (NRC) recommendation (552–3,200 IU vitamin D3/kg food dry matter) for maintenance of adult dogs (15). The food label indicated that it contained 21% crude protein, 9% fat, 3% fiber, and 10% multivitamins and minerals.

The dogs were monitored twice daily for clinical symptoms. Throughout the study, no clinical signs were observed. During nearly all visits, no leftover food was found. The dogs were exercised for approximately 45 min each day. Their weight was recorded weekly, and no significant changes in body weight were noted throughout the study.

After two weeks of an adaptation period, all 8 dogs received vitamin D3 supplements in a commercial form (D-Vigel 1,000^®^, Daana Pharma Co., Iran) at the dose of 50 IU/kg BW per day (13). The supplement was administered along with food and offered as a small treat. Blood samples from the jugular vein were taken from each dog on days 0, 14, 28, and 42 of the study, with a maximum volume of 5 mL collected from each animal. Sampling was performed by using plain vacutainer tubes for determination of serum biochemical parameters. Blood samples were held at room temperature for about 30 min for complete clot formation. The samples were then rimmed and centrifuged at 450 rpm for 10 min. Harvested sera were kept at −20 °C until use.

The ethics committee for biomedical research at Shiraz University approved the animal study in accordance with guidelines that align with Directive 2010/63/EU, which focuses on safeguarding animals utilized for scientific objectives.

### Serum thyroid hormones and TSH assays

To assay serum T4, fT4 and T3, commercial competitive ELISA kits prepared by DiaZist, Tehran, Iran, were used. The detection limit of T4 ELISA kit was 0.3 μg/dL. For fT4 and T3 the detection limits were 0.1 ng/dL and 0.1 ng/mL, respectively. The inter assay and intra assay CVs for all kits were <5%.

An ELISA kit prepared by Nouyan Negin Parsian Co., Tehran, Iran was used to assay fT3. The detection limit of the kit was 0.52 pg./mL. The kit worked by competitive ELISA method. The intra assay and inter assay CVs of the kit were <10 and <15%, respectively.

For all kits the absorbance was read at 450 nm wave length with a differential filter between 620–630 nm.

A canine thyroid stimulating hormone (cTSH) ELISA kit (Padgin Teb, Tehran, Iran) was used to determine serum TSH level. The kit worked on the basis of sandwich ELISA method with the detection limit of 0.3 mIU/L. Inter assay and intra assay CVs of the kit were <12 and <10%, respectively. The absorbance was read at 450 nm.

All procedures were performed according to the instructions of manufacturers.

### Data analysis

Data related to each time point were presented as mean±SD. Data analysis was performed by repeated measures one-way ANOVA followed by Tukey’s multiple comparison test. *p* < 0.05 was considered as the level of significance for all comparisons. Data analysis and preparation of graphs were performed by GraphPad Prism 6 software.

## Results

### Serum T4 and fT4 levels

Six week vitamin D3 supplementation had a statistically significant effect on serum T4 levels [*F* (1.89, 13.2) = 8.39 and *p* = 0.004]. A slight increase was observed in serum T4 level on day 14 post vitamin D3 supplementation as compared to day 0 (*p* > 0.05). Serum T4 level on days 28 and 42 post supplementation was significantly higher than the baseline level (day 0) (*p* < 0.05 and *p* < 0.01, respectively), while no significant difference was observed between day 28 and 42 (*p* > 0.05).

**Figure 1:**
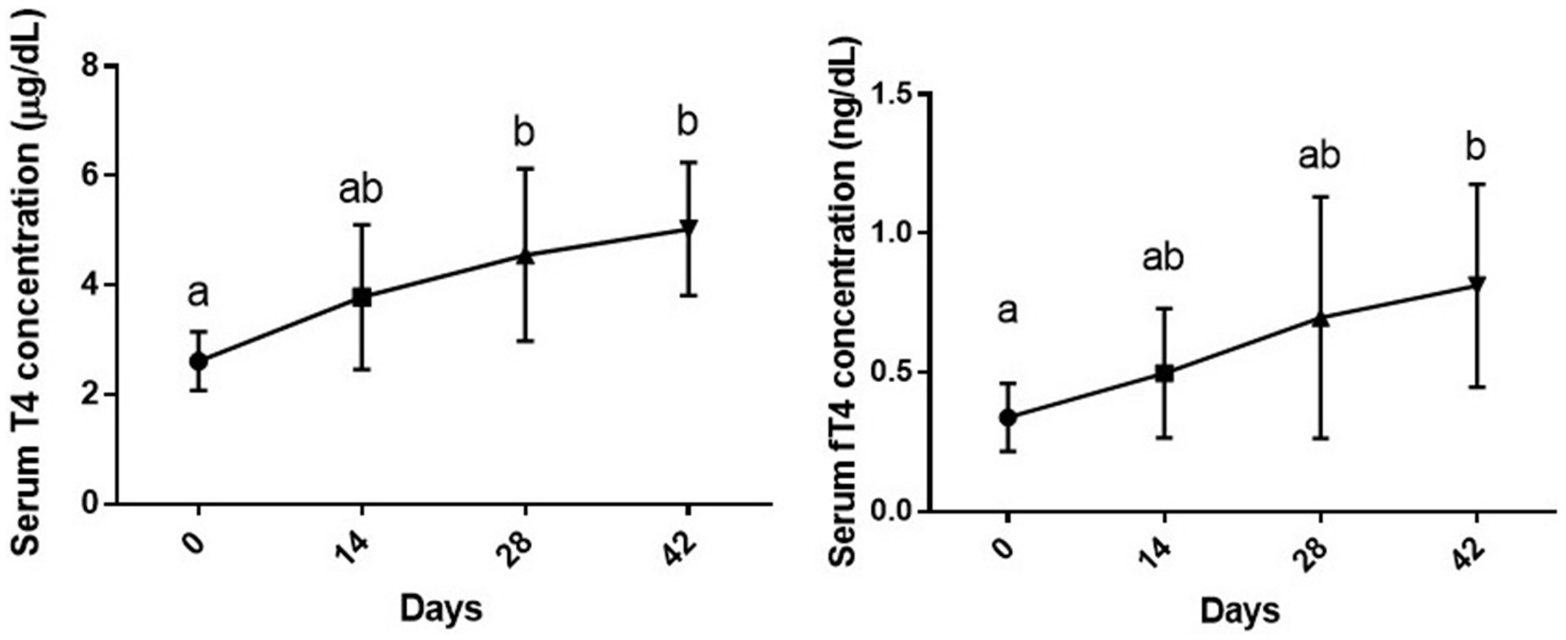
Serum levels of total and free T4 (mean ± SD) of dogs (*n* = 8) in different sampling time points following vitamin D3 supplementation. Values in time points with no common superscript letter indicate significance at *p* < 0.05 (Tukey’s multiple comparison test).

There was also a statistically significant effect of duration of supplementation on serum fT4 levels, [*F* (1.63, 11.4) = 12.53, *p* = 0.014]. Although slight increases were observed in this parameter on days 14 and 28 of the study as compared to day 0, the difference was only significant between day 0 and 42 (*p* < 0.05) (Figure 1).

### Serum T3 and fT3 levels

**Figure 2:**
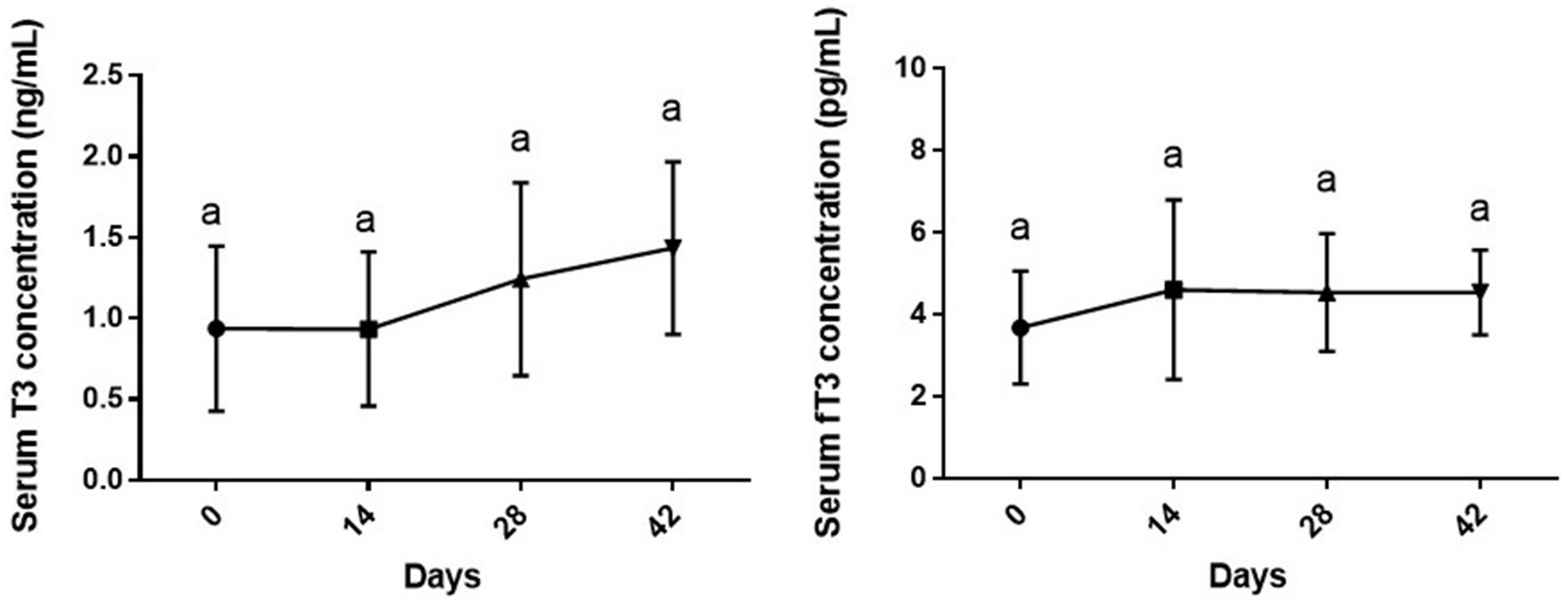
Serum levels of total and free T3 (mean ± SD) of dogs (*n* = 8) in different sampling time points following vitamin D3 supplementation. Similar superscript letters indicate no significance at *p* < 0.05 (Tukey’s multiple comparison test).

As shown in Figure 2, changes in serum levels of T3 [*F* (3, 28) = 1.71, *p* = 0.187] and fT3 [*F* (1.574, 11.02) = 1.38, *p* = 0.283] of dogs during the study period were not statistically significant compared to day 0 or other sampling time points (*p* > 0.05).

### Serum T3/T4 and fT3/fT4 ratios

**Figure 3:**
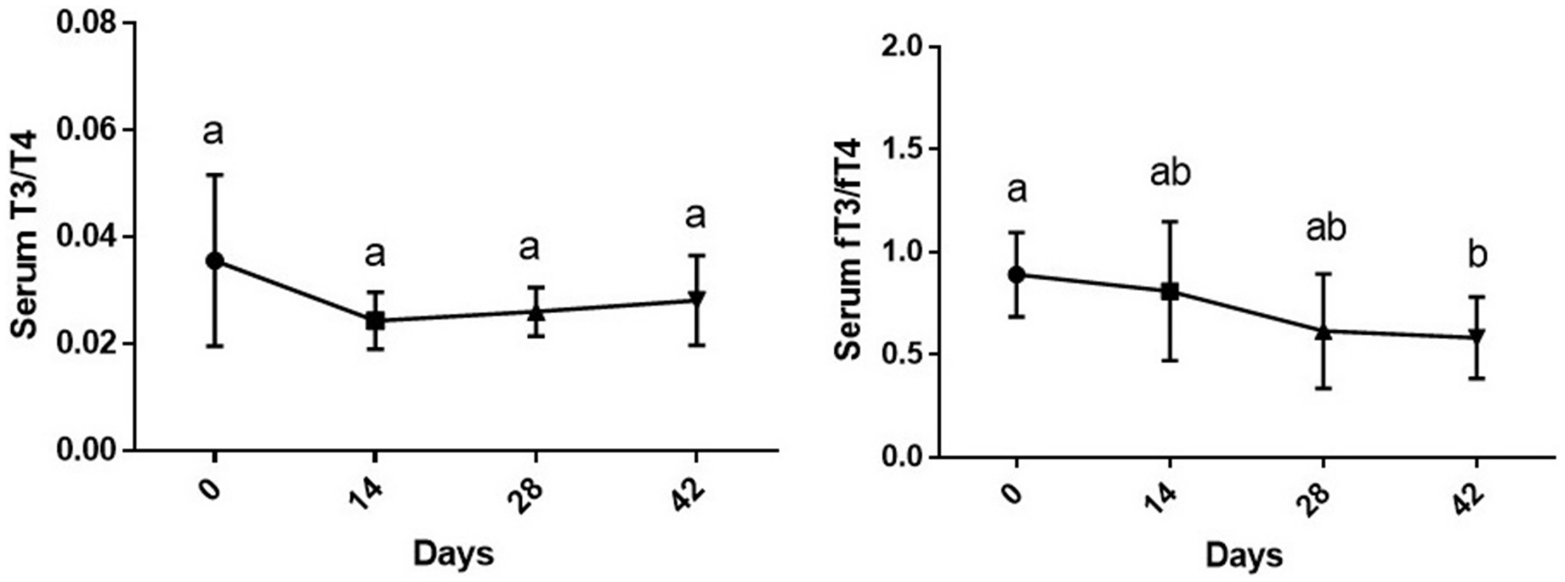
Serum T3/T4 and fT3/fT4 ratios (mean ± SD) of dogs (*n* = 8) in different sampling time points following vitamin D3 supplementation. Values in time points with no common superscript letter indicate significance at *p* < 0.05 (Tukey’s multiple comparison test).

While the changes in serum T3/T4 during time were not statistically significant (p > 0.05), changes in fT3/fT4 ratio due to supplementation of vitamin D3 showed a downward significance during study period [*F* (1.77, 8.87) = 5.18 and *p* = 0.035]. The fT3/fT4 ratio on day 42 of the experiment was significantly lower than day 0 (*p* < 0.01) (Figure 3).

### Serum TSH levels

**Figure 4:**
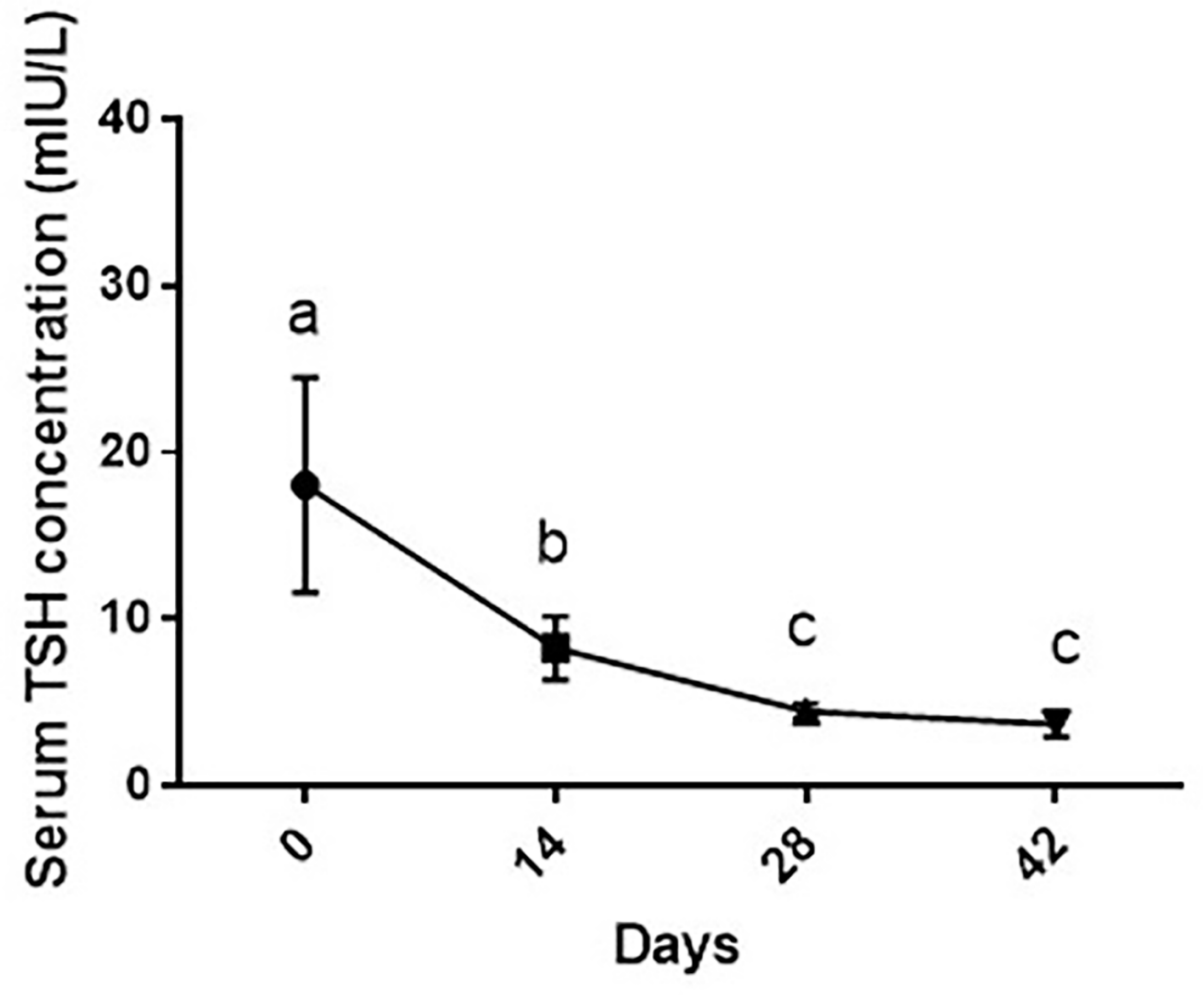
Serum TSH levels (mean ± SD) of dogs (*n* = 8) in different sampling time points following vitamin D3 supplementation. Values in time points with no common superscript letter indicate significance at *p* < 0.05 (Tukey’s multiple comparison test).

As shown in Figure 4, changes in TSH levels due to supplementation of vitamin D3 showed a significant decrease during the study period [*F* (1.17, 7.02) = 26.4 and *p* = 0.001]. Serum TSH level of dogs was statistically lower on days 14 (*p* < 0.05), 28 and 42 than day 0 (*p* < 0.01 for both comparisons). On day 14, serum TSH level was significantly higher than days 28 and 42 (*p* < 0.05 and *p* < 0.01, respectively) with non-significance between days 28 and 42 (*p* > 0.05).

## Discussion

In the present study, we observed that administration of a vitamin D3 supplement to healthy dogs is associated with a time-dependent increase in serum T4 and fT4 levels as well as decreased fT3/fT4 ratio and TSH concentration without affecting T3 or fT3 levels. The values for serum T4, fT4, fT3/fT4 ratio and TSH, were statistically the same between days 28 and 42. This shows that the effect of vitamin D on these parameters was more profound after 1 month of administration at the dose of 50 IU/kg and then the magnitude of the effect only slightly increases. Unfortunately, we are unable to clarify the exact reasons behind this observation based on our study’s results, as various regulatory mechanisms may be at play for both thyroid hormones and vitamin D in this regard.

In this current research, the observed rise in serum T4 and fT4 levels after vitamin D3 supplementation appears to occur due to an effect at the thyroid gland level not pituitary, as evidenced by the lowered TSH concentrations, likely due to the negative feedback of thyroid hormones on the hypothalamus-pituitary-thyroid axis. In fact, the main mechanism that regulates TSH secretion is the negative feedback effect of thyroid hormones in their free or unbound forms. When the blood concentrations of T3 and T4 increase, the production and release of TSH and TRH from the hypothalamus and pituitary gland are suppressed. Although T3 is taken up by cells more quickly and produces effects faster than T4, the majority of T3 is generated from T4 in target tissues by the action of iodothyronine deiodinases (16). Therefore, the decreasing trend in TSH levels as observed in the study might be due to inhibitory effect of thyroid hormones.

It is well-established that the effects of 1,25-dihydroxyvitamin D3 as the biologically active metabolite of vitamin D are mediated through the nuclear vitamin D receptor (VDR) (17). The presence of this receptor and the enzymes responsible for activating and catabolizing vitamin D are shown in the cancerous and non-cancerous thyroid tissue of humans (18–20). Also the presence of VDR is shown in normal follicular thyroid cells of rats (21).

Even though we could not locate a related study involving dogs, Šošić-Jurjević et al. (21) found that administering orchidectomized rats 200 IU/kg of vitamin D3 for three weeks resulted in an increased utilization of stored colloid for the release of thyroid hormones into the bloodstream compared to non-supplemented orchidectomized rats; however, these researchers did not find any significant variations in the levels of circulatory T4 or TSH in the supplemented animals (21). It still needs to be determined in future research whether the increase in serum levels of T4 and fT4 observed in the current study is associated with accelerated release and/or production of thyroid hormones. Moreover, further advanced research (such as assessing the influence of breed on vitamin D metabolism or its impact on thyroid function) should be conducted in the future.

The T3/T4 ratio is a simply calculated index reflecting thyroid function and the action of hormones on the tissues (22). Despite, the increased level of T4, we only found a slight decrease in T3/T4 ratio during the study which was probably related to subtle increases in T3 levels.

As stated previously, despite to the observed rise in T4 and fT4 levels, no significant change was detected in T3 or fT3 concentrations of dogs after vitamin D3 supplementation. The T3 hormone is more potent than T4 and a substantial part of T3 is made by the action of deiodinases in the thyroid gland or in peripheral tissues on T4 (23). In fact, the T4/T3 ratio stored in the canine thyroid is 12/1 while the ratio of secreted products is 4/1; moreover, 40–60% of T3 in the dog is derived from extra thyroidal deiodination of T4 (2). The conversion of T4 to T3 is a critical regulatory step in peripheral tissues to acquire T3 which properly meets their demands (23). Unfortunately, we did not assay the possible effect of vitamin D3 on the activity of deiodinases in the current study. Type 1 (D1) and type 2 (D2) deiodinases transform the prohormone T4 into the active hormone T3 by means of outer ring deiodination; conversely, type 3 deiodinase (D3) inactivates T4 into reverse T3 (rT3) and converts T3 into T2 via inner ring deiodination (24). Limited knowledge is available on the effects of vitamin D on the activity of deiodinases. In a study performed by Alrefaie and Awad (25) supplementation of vitamin D3 in diabetic rats resulted in a notable rise in mRNA expression of the D2 enzyme in both the liver and brain, alongside elevated serum levels of fT3 and decreased fT4 concentration, with no significant alterations in the mRNA expression of D2 in the heart, bone, muscle, and kidneys when compared to diabetic non-supplemented animals.

Interestingly, the fT3/fT4 ratio represents the degree of transformation from T4 to T3, which is affected by the activity of deiodinase, this helps in understanding the sensitivity of peripheral tissues to thyroid hormones (26, 27). An inverse U-shaped relationship is found between fT3/fT4 and serum vitamin D levels in humans (26). In the present study, we observed appreciably decreased fT3/fT4 ratio after 42 days of vitamin D3 supplementation to dogs compared to baseline level. This shows a probable decrease in transformation of T4 to T3 by deiodinases. In the present study, all included dogs were euthyroid [(serum T4 levels of no dog were less than 0.8 μg/dL and all dogs showed TSH levels <0.6 ng/mL (33.5 mIU/L) at the beginning of the study (day 0))] (3, 28). Consequently, this observation may be attributed to homeostatic regulation of the activity of deiodinase enzymes based on the precise needs of specific peripheral tissues. The situation is different in hypothyroid states. Therefore, the effect of vitamin D administration on fT3/fT4 ratio in hypothyroid dogs is an important subject that needs to be addressed in future studies.

A fact that should be mentioned here is that in the current study, each dog received a total (from the diet and the supplement) of 2063 IU of vitamin D3 per 1,000 Kcal of dietary ME (the ME of the diet was 3,200 kcal/kg dry matter, the dry matter was about 90% and each 20 kg dog received 300 g food per day). The National Research Council (NRC) indicates that the safe upper limit of cholecalciferol for adult dogs is 800 IU per 1,000 kcal of ME. Consequently, the amount of vitamin D3 given to the dogs in this study surpassed the safe upper limit by a factor of 2.57.

In a study by Tryfonidou et al. (29), when growing dogs were administered excessive amounts of vitamin D (∼100-fold NRC-recommended) from 6 to 21 weeks of age, the active metabolite of vitamin D3 (1,25(OH)2D3) decreased by approximately 40% in high vitamin D-treated group compared to control group. This reduction was linked to an increased metabolic clearance rate in the high vitamin D group versus the control, along with a lack of the anabolic effect of PTH on the synthesis of 1,25(OH)2D3. This finding clearly illustrates the body’s tendency to mitigate the effects of excessive dietary vitamin D by decreasing the synthesis of its active metabolite. The authors observed no clinical sign of vitamin D toxicity; however, excessive non-toxic supplementation of vitamin D3 led to reduced bone remodeling. Consistently, in another study by Jewell and Panickar (30), feeding adult dogs a diet with 9992.5 IU/kg dry matter (approximately 3.12 times the highest recommendation by the NRC for adult dogs) over a duration of 6 months did not result in any noticeable negative effects.

On the contrary, in a study by Spangler et al. (31), dogs given excess vitamin D (20,000 or 40,000 IU/kg of body weight daily for 1 to 3 weeks) showed characteristic nephropathy associated with vitamin D toxicosis or hypercalcemia. These amounts are about 225 and 450 times the amount we used in the present study (~90 IU/kg BW in our study).

Collectively, it appears that, significantly elevated concentrations of vitamin D, compared to the safe upper limit suggested by the NRC, may be correlated with discernible negative clinical manifestations in canines. Nevertheless, the duration of supplementation (periodical versus lifelong) might play a very important role in this regard. Additional variables such as age, health status, breed, and so forth, may play a significant role in this context and need to be addressed in future studies. Corbee (11) suggests more research are necessary to ascertain the precise intervals of vitamin D requirement in dogs and cats.

One limitation of our research was the small sample size and the study’s design, which involved a self-controlled approach without a distinct control group, especially considering the relatively lengthy duration of the study. Additionally, the failure to assess various forms of vitamin D metabolites in serum and their potential association with thyroid hormones, along with the lack of determining the presence of thyroglobulin autoantibodies (TgAA) in dogs, are further limitations of the study.

In conclusion, vitamin D supplementation to healthy dogs is associated with a time-dependent change in thyroid hormone profile (increased serum T4 and fT4 levels); these effects are probably mediated at the thyroid gland level as shown by the negative feedback on serum TSH concentrations. These findings pave the road for future more sophisticated studies on the plausible effects of this vitamin on thyroid function of hypothyroid dogs.

## Data Availability

The raw data supporting the conclusions of this article will be made available by the authors, without undue reservation.
